# Medicinal plants in the treatment of urinary tract malignancies during the Araboislamic period (7^th^- 14^th^ century AD)

**DOI:** 10.1080/2090598X.2022.2077002

**Published:** 2022-05-18

**Authors:** Achillefs Drogosis, Charalampos Mamoulakis, Emmanuel Chrysos, Gregory Tsoucalas, Spyros N. Michaleas, Marianna Karamanou

**Affiliations:** a Department of History of Medicine and Medical Deontology, Medical School, University of Crete, Crete, Greece; b Department of Urology, University General Hospital of Heraklion, University of Crete, Medical School, Heraklion, Crete, Greece; c Department of General Surgery, Medical School, University of Crete, Crete, Greece; d Department of History of Medicine and Medical Ethics, Medical School, National and Kapodistrian University of Athens, Athens, Greece

**Keywords:** Herbal medicine, cancer, urology, islamic golden age

## Abstract

Arabic medicine, or Arab-Islamic, mainly refers to all developments achieved in the Age of Khalifs, or the Golden Age of the Arab-Islamic civilization (ca 7^th^-14^th^ centuries AD). Arab scholars adopted ancient Greek medicine and soon understood the essence of the fatal disease known as cancer. They introduced various new types of cancer, distinguishing other entities like infection and proposed new methods of treatment, both surgical and non-invasive. Herbal medicine after Dioscurides and Galen bloomed in the Arabic world. Malignancy of the urinary tract was identified and a plethora of herbs were used to slow down its expansion. Moreover, herbal drugs were introduced to alleviate cancerous symptomatology. Avicenna introduced Hindiba, while known scholars like Abulcasis and Rhazes noted the benefits of garlic, onion, black seeds, pomegranate, olive oil as well as leaf and bread wheat. Arabian herbal medicine may still be beneficial in anticancer fight and mainly in the palliative medicine. It should be emphasized that almost 50% of the drugs administered today have their point of origin in the plants used in antiquity.

## Introduction

The medical renaissance during the Age of Khalifs (ca 7^th^ to 14^th^ century), in the Arabian world, marked the birth of a school which flourished and evolved during a period of about 7 centuries. In the crossroad of great civilizations, Arab physicians inherited a huge volume of medical knowledge from the Hellenic peninsula, Persian Empire and India. This treasure, an essence of practical medicine, philosophy and science was adopted, translated and ameliorated before being transmitted to Western Europe to provoke the genesis of the modern medicine. Pupils arrived from both the west and east territories of the world to the Arab capitals of Cairo, Baghdad and Damascus to receive the highest education in the local medico-philosophical schools [1].

Arab physicians in the Bagdad area were among the first few who separated pharmacology from medicine. Herbal kingdom was a huge resource apothecary for pharmaceutical preparations for almost all diseases to be confronted. In the early years of the Arabic medical evolution, poisonous potions were in the first raw on interest. It became something like tradition the changes in the royal hierarchies to be made by an administration of a kind of poison. Soon, ca 10th to 12th century, alchemy and chemistry began to search for antidotes and cures. The Assyrian Nestorian physician Yuhanna ibn Masawaih (ca 777–857 AD) was the first to compose a treatise in pharmacology, introducing herbal therapeutics, aromatotherapy and immunity awareness. Potions, poultices, ointments, compresses, pessary, pills, drugs, powders, were all used, while diet had been also practiced (dietetic regime containing specific herbs). Persian Muslim physician Abu al-Hasan Ali ibn Sahl Rabban al-Tabari (ca 808–870 AD) in his treatise Paradise of Wisdom dedicated some chapters in pharmacology. The methods of production were analyzed, while personalized administration was introduced. According to his opinion every drug should have been used for one and only one disease. Glass and ceramic containers were proposed, as well as specialized jars for eye drops [2,3].

Due to the expansion of the Muslims’ empire during the 8^th^ century AD, a new breed of medicine was born, the Greco-Arab. The works and thus the thesis of the Greeks entered the Arabian world and the treatises of Hippocrates, Galen, Plato, Aristotle and many more were translated into the most prolific scientific language of the era, the Arabian. Soon, numerous innovations in internal pathology, surgery and surgical tools, clinical trials in pets and experiments were introduced. However, herbal medicine continued the legacy of Hippocrates, Dioscurides and Galen and further advanced with the use of a rich variety of plants. The Islamic medicine was ready to conquer the world [4].

For the Arab physicians, cancer was a fatal disease. The superficial types could have been operated, as for the rest palliative medicine and drug administration was the golden choice. To test every possible cure, as some herbs contain toxic substances, they had introduced experimentation with animals. Every cure was firstly studied for possible toxicity and potential efficacy, followed by a limited use in humans before noted in medical texts and mass administration [2].

The Hellenic concept to confront cancer was to operate the superficial types like breast, or manipulate into the cavities that is in the mouth, ear, nose, vagina and rectum. Having in mind that the detailed topographic anatomy and the physiology of those organs were somewhere in the terra incognita. Moreover, the operations were limited for the most part in amputations to remove or cauterize the formations that could develop [5,6]. The theory of the 4 humors which was introduced by the Hippocratic School to explain the theory of cancer manifestation, noted that the excess of one, mainly the black bile was the cancerous trigger for malignancies. Malignant tumors had been widely referred and believed to mainly afflict the adults. Surgery, burned iron and mixtures (herbs, animal products, organic metals) were at the disposal of the ancient Hellenic medicine [7]. Although Arabs accepted those views, many prominent Muslim Persians and Arab scholars studied and penned about all known types of cancer inside their works, improving treatment and unfolding new paths in oncology. Abu Qasim Khalaf Ibn Abbas Al Zahrawi, known in the West as Albucasis or Zahravius (936–1013 AD) was the first to distinguish between acute kidney inflammation and kidney cancer [4]. Abu Ali al-Husayn ibn Abd Allah ibn Sina, Latinized Avicenna (ca 980–1037 AD) proposed the early radical excision of the diseased tissue with amputation and removal of veins surrounding the growth, or cauterization if necessary [8]. The goal to firstly halt the growth and expansion of the tumor had been also realized by Abu Bakr Mohammad Ibn Zakariya Razi, Latinized as Rhazes (865 to 925 AD) and Abulcasis [9]. Avicenna had also proposed another pioneering for the era idea. He had encountered a case of a breast malignancy, a female patient who, after a radical excision, suffered from a second tumor in the other breast, leading him to the hypothesis that cancerous material advanced from the primary afflicted breast. The concept of cancer metastasis was established after an ingenious remark [10]. He was also among the many other Arab and Muslim scientists who introduced numerous new recipes about herbs and their potential medical efficacy and safety [4].

The tumor regression, ulcer treatment, pain palliation, thus a holistic approach of a patient suffering from the fatal disease of cancer was mainly based on herbs. This historical vignette presents herbs, mixtures, poultices and drugs used towards those purposes by the Arabs during their golden age.

## Herbal diet as treatment for cancer

**Figure 1.**
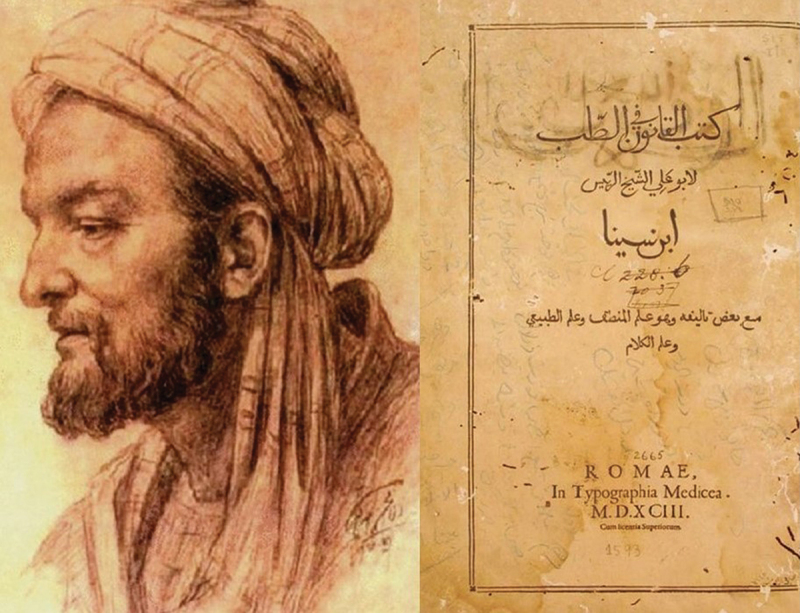
A portrait of Abu Ali al-Husayn ibn Abd Allah ibn Sina (Avicenna), Krueger HC, Avicenna’s poem on medicine. Springfield, Illinois, Charles C Thomas, 1963;p 52a (left side). The Canon of Medicine in Arabic which was published in Rome in 1593 (right side).

**Figure 2.**
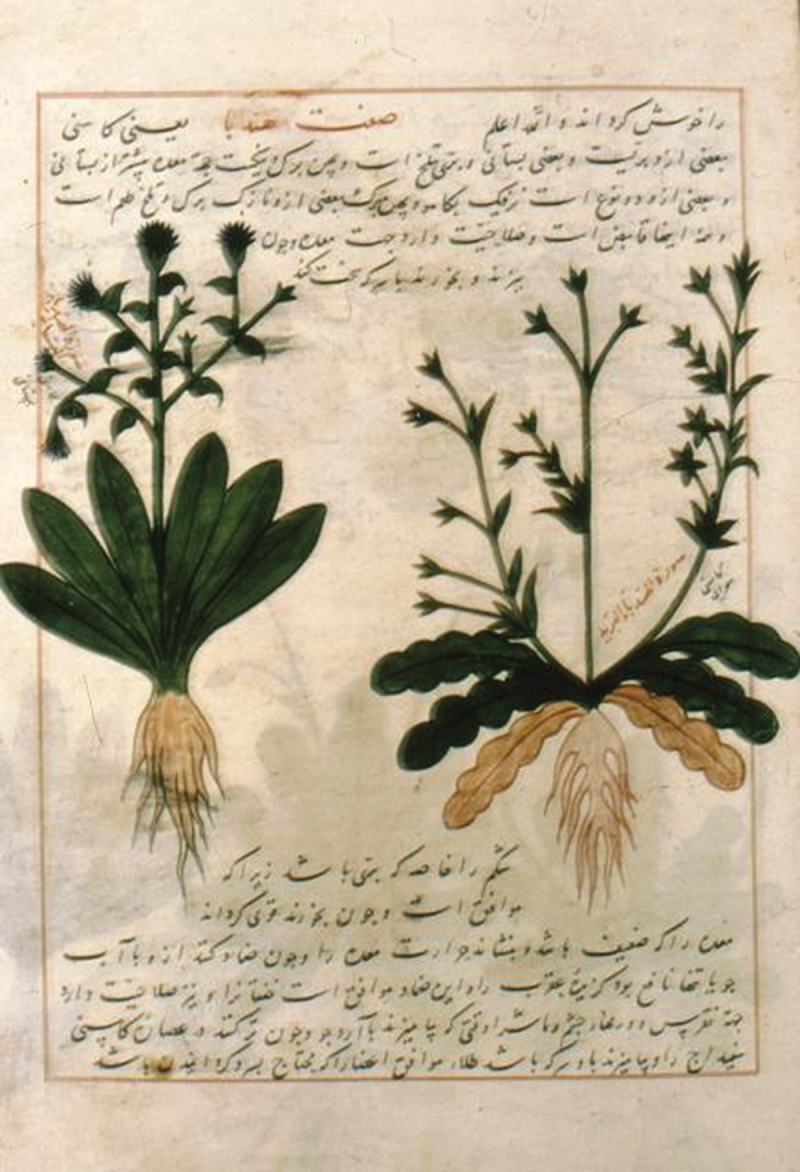
The cultivated and the uncultivated kinds of hindiba illustrated in Dioscurides Materia Medica translated by Abdullah el-Huseyin b. Ibrahim el-Natili. Topkapi Museum Library, No 2127.

Arabs and Muslim people adopted the concept of diet from the ancient Greeks. The Prophet Mohammad (Peace be upon him) himself noted that the diet is the source of health. Avicenna also emphasized for a thorough concentration upon a correct-healthy diet which should reinforce body strength and should prevent event cancer progression. Grapes, citrus, melon, squash, figs dates, honey, olive oil, black seed consumed alone as proposed in the holy ‘Quran’ and ‘Hadith’ by the Prophet (Peace be upon him). They were also consumed in mixtures as proposed in the ‘Canon of Medicine’ by Avicenna [Figure 1] ‘using mineral smears containing millstone dust and whet-stone dust, stone pounder for aromatics and black head stone moisturized with rose oil and coriander water poured on pounder, or dressings with well pounded verjuice’. All the above could be helpful for the clash between human immune system and tumorous growth. Fox-grape water, kernels of common wheat, rose oil, aloes in mixtures with white lead, scoria, crab, clay and water were also used [4]. Moreover, Avicenna introduced the chicorium intybus, an herbal compound drug with anticancer properties [Figure 2] [11]. Hilal Said and his colleagues in 2010 noted the 6 most used plants of the Arabian herbal medicine to confront cancer, namely garlic, onion, black seeds, pomegranate, olive oil and leaf and bread wheat [4]. All those herbs or herbal products were generally used as agents to control malignancy expansion, including those of the urinary tract, as cancer itself was considered in antiquity, by all great civilizations, as an eventually fatal disease [12].

## Medicinal plants to confront symptomatology of the urinary tract malignancies

**Figure 3.**
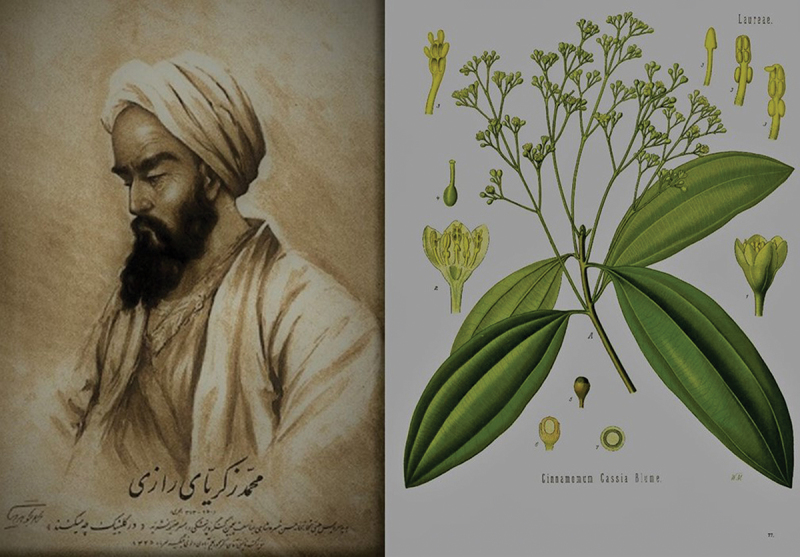
A portrait of Abu Bakr Mohammad Ibn Zakariya Razi (Rhazes), Welcome Library (left side). Cassia in Köhler’s Medizinal-Pflanzen (Medical Plants), 1887 (left side).

Although a variety of medicinal plants were used in general against cancer, a plethora of them were administered to palliate cancer symptomatology. Especially for the urinary tract glycyrrhiza-liquorize was used for the ulcerated kidneys (not in all cases the ulceration meant cancer), daucus-carrot, erythrodanum-madder and cinnamomum-cinnamon had been known to absorb substances and purge kidneys, while viola-violet, cucurbita sylvestris-colocynth, lapis lazuli-cyanus, opium-poppy, petroselinun-stone parsley and tragacantha-tragacanth gain their place as pain killers. Finally, hyssopum-hyssop was used to ameliorate hardness of the kidneys, a probable tumorous effect [13]. Avicenna had also proposed linum usitatissimum-flax, pinus brutia-pine and corylus-hazel as analgesics [14]. More anodynes were suggested by Rhazes who mentioned prunus amygdalus-almond, prunus cerasus-cherry and cassia-East Asian evergreen tree [Figure 3] [15].

The plants and their products proposed by the Arabs during their golden civilization age endured time. The holistic cancer management requires a combination of herbal medicine and modern drugs [16], while traditional herbal medicines provide a remarkable source for the half of the newly developed drugs [4]. Nowadays, complementary medicine, including herbal products, has a significant role in culture-centered care for cancer patients in the Middle East area. However, the majority of the modern Arab therapists have difficulties to propose a parallel therapeutic approach and the great legacy of their ancestors need to be reminded to be once more in vogue [17].

## Conclusion

Herbs were used by the Arabian school of medicine to prevent tumor genesis, malignancy proliferation and pathological symptoms. In theory, as cancer evolves over a long period of time, all agents that inhibit or retard one or more of its stages, as some herbs do (products and metabolites), could affect the overall course of the disease. Medical treatises of the past present a broad source of information concerning malignancy of the urinary tract, information which may reach contemporary physicians to enhance their effort in the anticancer war. Not all the herbs mentioned are validated in scientific laboratories and their efficacy and biochemical action should be thoroughly studied. However, herbal medicine constitutes a significant branch of the palliative medicine and the Arabs lived in the age of Khalifs were among those who established it.
